# Exploring the transformative influence of neuroplasticity on stroke rehabilitation: a narrative review of current evidence

**DOI:** 10.1097/MS9.0000000000001137

**Published:** 2023-08-07

**Authors:** Nicholas Aderinto, Muili O. AbdulBasit, Gbolahan Olatunji, Temilade Adejumo

**Affiliations:** a Department of Medicine and Surgery, Ladoke Akintola University of Technology, Ogbomoso; b Department of Medicine and Surgery, University of Ilorin, Ilorin, Nigeria

**Keywords:** neuroplasticity, rehabilitation, stroke, transformation

## Abstract

This review aims to assess the role of neuroplasticity in facilitating stroke recovery and identify the challenges and limitations associated with its implementation. A comprehensive literature search was conducted to identify relevant studies, which were meticulously evaluated to determine the potential solutions for effectively harnessing neuroplasticity. The results indicate that neuroplasticity holds significant promise in stroke rehabilitation; however, individual variability in response to interventions, timing and duration of interventions and sociocultural and clinical factors pose challenges. Tailoring interventions to individual patient characteristics is crucial for optimising the impact of neuroplasticity. Despite challenges and limitations, the transformative potential of neuroplasticity in stroke rehabilitation is undeniable. The abstract concludes by emphasising the importance of a comprehensive understanding of individual variability, optimising intervention timing and duration and considering sociocultural and clinical factors. Future research and clinical practice should prioritise personalised interventions and interdisciplinary collaborations to fully exploit the vast potential of neuroplasticity in stroke recovery.

## Introduction

Stroke continues to be a significant public health concern, ranking as the second-leading cause of mortality and the third-leading cause of mortality and disability combined, as measured by disability-adjusted life-years (DALYs) lost worldwide^1^. The economic impact of stroke is also substantial, with the estimated global cost surpassing US$721 billion, equivalent to 0.66% of the global gross domestic product^1^. Over the course of nearly three decades, from 1990 to 2019, the burden of stroke has witnessed a substantial increase in absolute numbers, including a 70.0% rise in incident strokes, a 43.0% increase in stroke-related deaths, a 102.0% rise in prevalent strokes and a 143.0% surge in DALYs^1^. Notably, the majority of the global burden of stroke, accounting for 86.0% of deaths and 89.0% of DALYs, is concentrated in lower-income and lower-middle-income countries^1^. Furthermore, in low-income countries, the impact of stroke is often exacerbated by limited access to specialised healthcare services, including stroke units, rehabilitation facilities and advanced technologies that promote neuroplasticity-driven recovery^2^. These resource disparities pose significant challenges for individuals seeking optimal stroke rehabilitation outcomes.

Traditional approaches to stroke rehabilitation have predominantly centred on facilitating functional recovery through compensatory strategies to alleviate the consequences of impairments rather than addressing their underlying causes^3^. However, a growing realisation within the scientific and medical communities has underscored the extraordinary transformative potential embedded within neuroplasticity. This recognition has prompted a paradigm shift in stroke rehabilitation, emphasising the harnessing of neuroplasticity to facilitate functional recovery and promote substantial and enduring improvements in long-term outcomes for stroke survivors.

Fundamentally, neuroplasticity encompasses the brain’s remarkable capacity to reorganise its structure and function in response to diverse internal and external stimuli^4^. This complex phenomenon involves a complex interplay of cellular, molecular and synaptic changes that enable the brain to adapt, learn and repair itself in the face of neurological damage caused by stroke. The inherent adaptability of the brain presents a fertile ground for developing innovative strategies that optimise recovery and restore lost functions.

Recent advancements in neuroimaging techniques, such as functional magnetic resonance imaging (fMRI) and diffusion tensor imaging, have offered invaluable insights into the neural correlates of neuroplasticity^5^. These sophisticated imaging modalities enable the visualisation and quantification of the structural and connectivity changes occurring in the brain during recovery. The transformative influence of neuroplasticity on stroke rehabilitation spans various domains. Motor rehabilitation, for example, has witnessed a shift from repetitive task-oriented training to more adaptive and intensive therapies that actively promote neuroplastic changes^6^. Innovative interventions, including constraint-induced movement therapy (CIMT) and virtual reality (VR)-based training, capitalise on the brain’s plasticity to facilitate motor recovery by stimulating the formation of new neural pathways and enhancing connectivity between damaged and healthy brain regions^7^. Likewise, cognitive rehabilitation has embraced the potential of neuroplasticity. Cognitive training programs, complemented by noninvasive brain stimulation techniques such as transcranial magnetic stimulation and transcranial direct current stimulation (tDCS), enhance cognitive functions by fostering neuroplastic changes in relevant brain networks^8^. These interventions promise to improve stroke survivors’ attention, memory, executive functions and overall cognitive performance.

This narrative review explores the transformative influence of neuroplasticity on stroke rehabilitation by examining current evidence from studies and rehabilitation interventions. It provides an overview of neuroplastic changes following stroke, explores approaches leveraging neuroplasticity for motor and cognitive rehabilitation, discusses limitations and challenges in clinical practice and identifies areas for future research. By harnessing the transformative potential of neuroplasticity, this review aims to improve outcomes and quality of life for stroke survivors.

## Methodology

A comprehensive literature search was conducted across electronic databases, including PubMed, Embase and the Cochrane Library, to identify relevant studies exploring the role of neuroplasticity in stroke rehabilitation. The search strategy utilised appropriate keywords and Medical Subject Headings (MeSH) to retrieve articles published in English from 2000 to April 2023.

The inclusion criteria encompassed original research articles, reviews and meta-analyses that investigated the impact of neuroplasticity on stroke recovery and rehabilitation, focusing on both animal models and human participants. Exclusion criteria included studies with insufficient data, case reports, editorials, conference abstracts and non-English language publications. A narrative synthesis approach was employed to summarise and analyse the findings from the included studies, organising the results thematically to highlight the challenges, limitations and potential solutions associated with harnessing neuroplasticity for stroke rehabilitation. The total number of articles reviewed was 51 after applying the inclusion and exclusion criteria.

## Understanding neuroplasticity

Neuroplasticity encompasses the brain’s remarkable capacity to adapt and reorganise its structure and function in response to various stimuli, including environmental changes, learning experiences, developmental processes and the aftermath of strokes or traumatic brain injuries^4^. This inherent quality is critical for the brain’s ability to adjust and recover from such insults. Among the mechanisms underlying neuronal regeneration and collateral sprouting, synaptic plasticity and neurogenesis play prominent roles (Table 1).

**Table 1:** Key neuroplastic changes associated with stroke rehabilitation

Neuroplastic changes	Description of changes	Neural structures involved
Dendritic remodelling	Structural changes in dendrites, including sprouting and arborisation	Affected and unaffected brain regions
Synaptic plasticity	Strengthening or weakening of synapses based on activity and experience	Neurotransmitter systems, cortical and subcortical regions
Cortical reorganisation	Changes in cortical maps and functional organisation of brain regions	Motor and sensory cortices, association areas
Neurogenesis	Generation of new neurons in specific brain regions	Hippocampus, subventricular zone
Axonal sprouting	Formation of new connections or sprouting of existing axons	Corticospinal tract, other neural pathways

Synaptic plasticity allows neurons to modify the strength of their connections in response to activity, facilitating vital processes like memory formation and learning^9^. Contrary to previous beliefs, neurogenesis has been shown to occur in the adult brain, contributing to its regenerative capabilities^10^. The cytoskeleton, a crucial component in developing the central nervous system, plays a pivotal role in neuronal connectivity and synapse formation. Axonal growth cones integrate multiple signals to guide axonal development, with intracellular cues regulating this intricate process^11^. The dynamic regulation of actin filaments and microtubules in dendrites and dendritic spines impacts synaptic plasticity and spine morphology. Repellent cues influence synaptogenesis and synaptic plasticity while impeding axon regeneration following central nervous system injury. Although the interaction between dynamic microtubules and F-actin in dendritic spines remains incompletely understood, proteins such as debris and IQ motif-containing GTPase-activating protein (IQGAP) are implicated in coordinating their activities. Targeting the cytoskeleton presents a potential avenue for stimulating neuroregeneration after injury.

Axonal sprouting and dendritic branching are two other processes contributing to neuroplasticity. Axonal sprouting occurs when neighbouring neurons extend their axons to establish new connections with damaged or underdeveloped brain regions, facilitating restoring functional connections and compensating for lost neural pathways. Dendritic branching involves modifications to dendrites, the neuronal branches responsible for receiving signals from other neurons. These modifications encompass the growth of new dendritic spines and the elimination of existing ones, fostering the development of new connections and remodelling existing ones.

Functional remodelling of the brain is a remarkable phenomenon that enables compensation for lost function in cases of brain damage or sensory deprivation. In such situations, the brain can reconfigure its functional networks, allowing unaffected regions to take on the functionality of damaged or inactive areas. For instance, blind individuals may rely on their visual cortex to process other sensory inputs, such as language, demonstrating the brain’s adaptive capacity to maintain or restore functionality.

## Neuroplasticity-based rehabilitation modalities in stroke recovery

Following a stroke, the brain demonstrates remarkable restorative abilities through neuroplasticity. It enables the generation of new neurons, the establishment of fresh neural pathways and the modification of cellular structures in response to environmental changes. Neuroplasticity encompasses various mechanisms, including interhemispheric lateralisation, new connections by association between cortical regions within the injured area and the reorganisation of cortical representational maps^12^. Studies conducted on animal models provide compelling evidence that the most significant advancements in recovery occur within a limited timeframe of heightened neuroplasticity following a stroke. Notably, altered neural activity and connectivity, both in function and structure, have been observed in the perilesional and remote regions and the contralateral hemisphere^13^. These changes are believed to underlie the mechanisms responsible for spontaneous recovery. One notable change attributed to neuroplasticity is the modulation of local cortical structure and function, where the affected regions of the brain undergo adaptive modifications to compensate for lost function^14^. Additionally, neuroplasticity can lead to the modulation of brain regions distant from the injury site, indicating a network-wide reorganisation to support functional recovery^14^. Furthermore, a significant alteration occurs in the interaction between the ipsilesional and contralesional hemispheres as the brain adapts and redistributes functions across these regions^15^. Another aspect of neuroplasticity is the remapping of somatotrophic representation, involving the reorganisation of sensory and motor maps within the brain to accommodate changes in the body^16^.

Exploring neuroplasticity mechanisms in stroke survivors has heavily relied on noninvasive functional neuroimaging techniques, including PET and fMRI. PET enables the analysis of local perfusion and glucose metabolism changes, while fMRI detects variations in blood flow and oxygenation^5^. Using these methods in studies has yielded intriguing insights into brain activity patterns among stroke survivors. Studies employing PET and fMRI have revealed significant findings regarding brain activity in individuals who have experienced a stroke. During movements of the affected hand, increased brain activity has been observed in both the unaffected and affected hemispheres^17^. Recruiting additional brain regions in the unaffected hemisphere has been associated with better motor recovery. Longitudinal studies have demonstrated that changes in brain activity over time are closely linked to functional recovery. Initially, motor activity decreases shortly after a stroke, gradually increasing until reaching levels comparable to those in healthy individuals^18,19^.

Furthermore, fMRI connectivity studies have provided valuable insights into the functional connections between different brain areas in stroke patients. Reduced functional connectivity between the lesioned and unaffected hemispheres has been observed^20^. However, improved functional recovery in later stages correlates with establishing functional connections between specific brain regions following a stroke. Analyses of effective connections have demonstrated that the unaffected hemisphere can either facilitate or suppress activity in the lesioned hemisphere, depending on the severity of motor impairment^21^.

The efficacy of various activities in improving neuroplasticity in stroke patients has been demonstrated through animal and human model studies (Table 2). Among these activities, physical therapy has shown promising results. Landsmann *et al*.^22^ conducted a study involving eight stroke patients who underwent physical therapy and engaged in various physical activities. Neurophysiological assessment using MRI scans revealed significant neuroplasticity changes, as evidenced by increased activations in the brain’s gyrus and frontal lobe regions. These findings indicate that physical therapy induces neuroplastic changes that contribute to functional recovery in stroke patients. A recent study has highlighted the potential of aerobic exercise in enhancing the capacity of the motor system for neuroplasticity through the upregulation of neurotrophins, including brain-derived neurotrophic factor^23^. This study suggests that aerobic exercise can effectively augment neuroplastic changes. Furthermore, another study explores the multifaceted role of physical exercise as a diagnostic, rehabilitation and preventive tool for stroke^24^. Evidence from animal studies has also provided valuable information on the effects of specific interventions on neuroplasticity in stroke recovery. For example, a recent study investigated the impact of voluntary exercise on spontaneous recovery in mice^25^. MRI scanning techniques examined functional connectivity and white matter integrity in the mice’s brains. The results demonstrated that voluntary exercise facilitated the recovery of myelin density, which is crucial for efficient neural communication. Additionally, exercise promoted increased functional connectivity and improved cerebral blood flow and vascular quality in the mice. These findings underscore the positive influence of exercise on neuroplasticity and suggest its potential as a therapeutic approach for stroke recovery.

**Table 2:** Table comparing different neuroplasticity-based interventions in stroke recovery

Intervention type	Description of intervention	Targeted neural mechanisms	Efficacy in stroke recovery
Constraint-induced movement therapy (CIMT)	Restricting the use of the unaffected limb to promote intensive use of the affected limb	Motor cortex reorganisation, synaptic plasticity	Improved motor function, increased use of affected limb
Physical therapy	Rehabilitation techniques involving exercises, stretches, and movements to improve motor function and mobility	Motor learning, neuroplasticity	Improved motor function, functional outcomes
Transcranial direct current stimulation (tDCS)	Noninvasive brain stimulation using a weak direct current to modulate neural activity in targeted brain regions	Modulation of cortical excitability, synaptic plasticity	Improved motor function, cortical reorganisation
Speech therapy	Targeted exercises and techniques to improve speech and language deficits resulting from stroke	Neuroplasticity in language areas, cortical reorganisation	Improved speech and language function
Brain–machine interface (BMI)	A direct connection between the brain and an external device, allowing individuals to control devices using their brain signals	Neuroplasticity, cortical reorganisation	Improved motor function, communication, and control of external devices
Brain–computer interface (BCI)	Similar to BMI, BCI enables communication and control of devices using brain signals, focusing on nonmotor functions	Neuroplasticity, cortical reorganisation	Improved communication, assistive technology control, cognitive function, and quality of life
Cell therapy	Transplantation of stem cells or progenitor cells into the brain to promote regeneration and functional recovery	Neuroregeneration, trophic support, modulation of neuroinflammation	Potential for improved motor and cognitive function, but further research is needed

In addition to physical therapy and exercise, CIMT has emerged as a novel method for improving neuroplasticity in stroke patients^26^. CIMT is known to promote motor recovery after stroke, but the exact mechanisms underlying its effectiveness are not yet fully understood. However, recent research has provided valuable insights into the potential neuroplastic changes induced by CIMT. A study investigated the neuroplastic effects of CIMT and found that it promotes structural neuroplasticity primarily oriented towards the contralesional hemisphere while eliciting bihemispheric functional neuroplasticity^27^. These findings suggest that CIMT can induce adaptive changes in the brain, contributing to motor recovery. Another study explored the profound plastic changes in the brain with the implementation of CIMT, although the precise mechanisms behind these changes remain fully elucidated^28^. Nevertheless, this study provided evidence of the significant impact of CIMT on promoting neuroplasticity in stroke rehabilitation. Collectively, these studies underscore the potential of CIMT to induce beneficial neuroplastic changes in stroke patients, both in terms of brain structure and function. However, further research is warranted to better understand the precise mechanisms through which CIMT exerts its effects on neuroplasticity. Moreover, CIMT has been found to enhance dendritic plasticity in both the ipsilateral and contralateral sensorimotor complexes and increase the expression of growth factors, contributing to restoring motor function in stroke patients.

tDCS is a noninvasive technique that has shown promise in facilitating stroke recovery by inducing neuroplasticity. The pathological processes following a stroke provide a valuable framework for investigating how tDCS promotes neuronal plasticity and facilitates functional recovery. In a study involving mice with focal ischaemia of the motor cortex, researchers examined the effects of bihemispheric tDCS on forelimb motor function recovery^29^. The study aimed to evaluate the behavioural outcomes and the underlying mechanisms associated with tDCS treatment. The study’s findings demonstrated the effectiveness of tDCS in promoting motor recovery. Additional studies have revealed that tDCS can stimulate the production of growth factors, including BDNF, which promotes neuroplastic changes associated with motor recovery^29,30^. Additionally, tDCS has been found to improve dendritic spine density and enhance functional connectivity between motor and somatosensory cortices, further supporting the induction of neuroplastic changes following stroke^29^.

Combining CIMT with tDCS has emerged as a promising approach to stroke rehabilitation. A recent clinical study conducted in 2023 explored the combined use of tDCS and CIMT in poststroke patients, specifically targeting motor and functional upper limb recovery^31^. The study demonstrated that patients who received this combined intervention showed functional improvement, attributed to the underlying neuroplasticity mechanisms triggered by the interventions. Using CIMT and tDCS as adjunctive therapies provides valuable insights into the role of neuroplasticity in stroke recovery. These interventions leverage the brain’s capacity to reorganise and adapt, leading to functional improvements in individuals affected by stroke. CIMT and tDCS restore motor function and overall functional recovery in stroke patients by enhancing dendritic plasticity, growth factor expression and functional connectivity.

An important study investigated the integration of tDCS with occupational and physical therapy, comparing it to therapy combined with sham stimulation. The results revealed notable improvements in performance on the Fugl-Meyer Assessment (FMA) and Wolf Motor Function Test (WMFT) when tDCS was utilised^32^. This innovative approach effectively facilitated neuroplasticity and significantly enhanced functional outcomes for stroke patients. Furthermore, the implementation of robotic hand exoskeletons has emerged as a promising avenue for fostering neuroplasticity among individuals in stroke recovery. A study compared the impact of an exoskeleton-based therapy on functional rehabilitation outcomes and cortical excitability to conventional rehabilitation methods^33^. The findings indicated that this novel therapy successfully promoted neuroplasticity and improved functional outcomes for stroke patients.

In a separate study, researchers explored multiple assessment methods for neuroplasticity, such as tDCS, electroencephalography (EEG)-based brain–computer interface (BCI) and neuroimaging (fMRI), alongside various robotic rehabilitation treatments^34^. The study demonstrated compelling evidence supporting the efficacy of robotic treatment targeting the upper limb in chronic stroke and cerebral palsy patients, improving motor function and facilitating neuroplasticity.

Speech therapy, as another valuable rehabilitative approach, has also garnered attention for its potential to promote neuroplasticity in stroke recovery. Through targeted exercises and activities that challenge the brain, speech therapy has proven effective in stimulating the formation of new neural connections, thereby improving overall brain function^35^. Moreover, applying motor learning principles to speech therapy has shown promise in enhancing poststroke recovery^36^. These studies highlight the diverse range of rehabilitative approaches that contribute to neuroplasticity in stroke patients.

Technological advancements have played a significant role in developing innovative approaches to enhance neuroplasticity in stroke recovery. The integration of artificial intelligence (AI), VR and telemedicine has opened up new avenues for delivering personalised and engaging interventions^37^. Among these approaches, BMI have emerged as a promising tool for guiding motor rehabilitation interventions, particularly in patients with limited or no residual movement. A recent review sheds light on the effectiveness of BMI technologies in facilitating neuroplasticity and promoting motor recovery following a stroke^38^. By capitalising on the brain’s inherent plasticity, BMI interventions offer the potential to induce reorganisation and rewiring of neural circuits, ultimately leading to improvements in motor function. These interfaces establish a direct connection between the patient’s brain and external devices, enabling control and manipulation of these devices through neural signals. The utilisation of BMI technologies in stroke rehabilitation has shown promising results, underscoring their capacity to drive adaptive changes in the brain that support functional recovery.

In addition to BMI, other pioneering techniques and therapies have emerged in neurorehabilitation. Cell therapy, for instance, involves the transplantation of stem cells or neural precursor cells, presenting significant potential for promoting the regeneration and repair of damaged neural tissue^39^. By introducing these cells into the affected areas, cell therapy aims to enhance the brain’s innate regenerative capabilities and facilitate neuroplastic changes contributing to improved motor function.

BCIs, akin to BMIs, have garnered considerable attention for their potential in stroke rehabilitation. BCIs enable individuals to interact with external devices or virtual environments using brain activity. Through the decoding and translation of neural signals generated during motor imagery tasks, BCIs provide a means for patients to control and manipulate virtual objects or prosthetic devices^40^. This intervention enhances motor function and stimulates neuroplastic changes in the brain, promoting overall improvements in motor performance. Furthermore, a systematic review and meta-analysis support the effectiveness of BCI training based on noninvasive EEG using motor imagery to improve functional recovery after stroke^41^. Individuals generate distinct EEG signals that can be decoded and utilised to control external devices or interact with virtual environments by engaging in mental imagery of specific motor tasks. This type of training enhances motor function and stimulates neuroplastic changes in the brain, contributing to overall improvements in motor performance.

## Factors influencing the effectiveness of neuroplasticity-based rehabilitation modalities in stroke recovery

Several sociocultural, clinical and genetic factors substantially influence neuroplasticity’s viability in stroke recovery. Sociocultural factors, including age, race and sex, have been demonstrated to impact the responsiveness to neurorehabilitation interventions. Social support and cultural beliefs have emerged as significant elements affecting the recovery process^42^. Moreover, a separate study has underscored the significance of experience and learning-dependent plasticity in stroke rehabilitation, leveraging insights from neuroscience^43^. Accordingly, comprehending the conditions that foster, facilitate and consolidate neuroplasticity is paramount in optimising stroke recovery.

Several factors associated with low-income countries can hinder neuroplasticity and impede the potential for functional recovery. Limited availability of trained healthcare professionals, including rehabilitation specialists, can result in delayed initiation of rehabilitation interventions, leading to missed opportunities for neuroplastic changes. Additionally, socioeconomic stressors, such as financial constraints, inadequate social support systems and cultural beliefs surrounding disability, may further hinder neuroplasticity processes. These factors can contribute to reduced motivation, increased stress levels and limited engagement in rehabilitation activities, all of which can adversely affect neuroplasticity outcomes. Similarly, environmental factors, such as limited access to assistive devices, adaptive equipment and rehabilitation technologies, can restrict opportunities for intensive and task-specific training that promote neuroplasticity. Inadequate infrastructure, including inaccessible environments and transportation barriers, can also hinder individuals’ ability to participate in community-based rehabilitation programs. Addressing the relevance of neuroplasticity in low-income countries requires a holistic approach. It involves advocating for improved healthcare infrastructure, increased availability of rehabilitation services and culturally sensitive interventions that promote patient engagement and motivation. It also necessitates community-based initiatives that empower individuals with stroke and their families through education, awareness and support networks.

Clinical factors play a pivotal role in determining the efficacy of interventions based on neuroplasticity. The specific type of stroke, poststroke complications and the precise rehabilitation strategies employed can profoundly influence treatment outcomes^44^. Tailoring therapeutic interventions to the individual needs and characteristics of stroke patients becomes imperative to optimise their recovery and capitalise on the benefits offered by neuroplasticity. Moreover, genetic factors contribute to interindividual variations in neuroplasticity and response to rehabilitation. Genetic variations can influence the brain’s plasticity capacity, recovery potential and treatment outcomes^45^. A comprehensive understanding of the genetic foundations of neuroplasticity in stroke recovery may facilitate the identification of patients who are more likely to benefit from specific interventions and inform the development of personalised treatment approaches.

Congenital diseases, such as cerebral palsy and genetic disorders affecting brain development, present unique challenges to neuroplasticity processes. These conditions can disrupt neural connectivity, impair synaptic plasticity and alter cortical reorganisation from early stages of brain development^46^. Consequently, individuals with congenital diseases often exhibit reduced neuroplasticity potential compared to those without these conditions. The impact of congenital diseases on neuroplasticity requires a nuanced understanding of the specific conditions and their underlying mechanisms. For example, cerebral palsy, characterised by motor impairments, can lead to abnormal muscle tone and a limited range of motion, affecting motor learning and reorganisation^47^. Genetic disorders affecting brain development often result in structural abnormalities, functional deficits and altered neurochemical processes that can influence neuroplasticity outcomes. Therefore, designing effective rehabilitation strategies for individuals with congenital diseases requires a multidisciplinary approach. Rehabilitation interventions should be tailored to address specific impairments, utilising techniques that promote neuroplasticity, such as task-oriented training, adaptive equipment and assistive technologies. Early intervention programs and comprehensive care coordination involving healthcare professionals, educators and families are essential for optimising neuroplasticity and facilitating functional improvements.

The timing of rehabilitation interventions is critical, as the brain exhibits heightened receptiveness to therapy during the acute and subacute stages following a stroke^44^. This pivotal period provides an optimal environment for neuroplastic changes and functional recovery. Early and uninterrupted rehabilitation endeavours prove indispensable in maximising long-term outcomes and harnessing the brain’s adaptive capacity. Neuroplasticity facilitates adaptive learning and skill acquisition, enabling individuals to enhance motor patterns and acquire new abilities. Interventions like mirror therapy, VR-based training and sensory integration exercises leverage the potential of neuroplasticity to promote functional recovery and engage the affected areas.

Pharmacological treatments also hold promise for augmenting neuroplasticity and facilitating stroke recovery. Certain pharmacological agents that target neurotransmitter systems, such as dopamine or serotonin, have exhibited the ability to influence synaptic plasticity and promote neuroplastic changes^48^. When used with rehabilitation techniques, these pharmacological approaches can enhance the brain’s restructuring capacity and facilitate the formation of new connections, thereby fostering greater functional recovery.

## Challenges and future directions

Neuroplasticity in stroke rehabilitation presents numerous challenges and limitations that demand thorough consideration to achieve optimal outcomes. A notable obstacle arises from the inherent variability in individual responses to neurorehabilitation, as each stroke survivor possesses a unique potential for neuroplasticity and exhibits varying recovery rates. This variability poses a daunting task in accurately predicting and effectively tailoring interventions.

The timing and duration of interventions also present challenges. A critical window of opportunity exists for neuroplastic changes and administering interventions either too early or too late may result in suboptimal outcomes. Determining the precise timing and duration of rehabilitation for each patient remains complex. Additionally, various individual factors can impact the viability of neuroplasticity in stroke recovery. Sociocultural factors, encompassing age, race and sex, may influence an individual’s response to neuroplastic changes. Clinical factors, such as stroke type, poststroke disorders and the specific rehabilitation strategies employed, can likewise wield significant effects on the ultimate outcomes. Therefore, comprehending and addressing these factors are paramount in tailoring interventions and optimising the potential of neuroplasticity-based rehabilitation.

Emerging technologies and approaches in neuroplasticity hold tremendous promise for enhancing stroke rehabilitation. Brain stimulation techniques, such as transcranial magnetic stimulation and tDCS, exhibit the potential to modulate neural activity and foster neuroplastic changes, thereby contributing to improved motor recovery. VR and AR present innovative tools in the field of stroke rehabilitation. VR creates immersive environments that facilitate motor learning and functional recovery, while AR enhances performance in everyday tasks and supports neuroplasticity-based rehabilitation.

Using robotics and exoskeletons as assistive devices in stroke rehabilitation is gaining increasing attention. These technologies enable precise and repetitive movements, promoting motor recovery and facilitating neuroplasticity. Customised assistance tailored to the individual needs of stroke survivors has the potential to maximise the prospects for neuroplastic changes. Future research and clinical practice should focus on optimising the impact of neuroplasticity in stroke rehabilitation. Personalised interventions based on individual characteristics, such as stroke type and severity, can enhance treatment outcomes. Using biomarkers and neuroimaging markers capable of predicting neuroplasticity potential may guide intervention selection and improve the development of patient-specific rehabilitation strategies.

Advancements in AI and ML hold promise for enhancing neuroplasticity-based rehabilitation. AI and ML algorithms can analyse vast datasets, providing personalised treatment recommendations based on patient characteristics and response patterns. Interdisciplinary collaborations among researchers, clinicians, engineers and technology developers play a vital role in advancing the field of neuroplasticity in stroke rehabilitation. These collaborative efforts facilitate the translation of emerging technologies and research findings into clinical practice, ensuring widespread implementation and optimising stroke recovery outcomes.

## Conclusion

The study of neuroplasticity’s impact on stroke rehabilitation holds great potential but faces challenges. Individual variability in responses, timing/duration of interventions and sociocultural/clinical factors complicate treatment. However, emerging technologies like brain stimulation, V/A reality and robotics offer promising avenues. Personalised interventions, understanding underlying mechanisms and interdisciplinary collaborations are key for optimising neuroplasticity’s role in stroke recovery. Future research and practice must focus on these areas to improve outcomes and enhance the lives of stroke survivors.

## Ethical approval

Ethical approval is not applicable for this review.

## Consent

Informed consent is not applicable for this review.

## Sources of funding

No external funding was received for this study.

## Author contribution

N.A.: conceptualization. All authors contributed to the writing of the first and final drafts.

## Conflicts of interest disclosure

The authors declare that they have no conflicts of interest.

## Research registration unique identifying number (UIN)

1. Name of the registry: not applicable.
2. Unique identifying number or registration ID: not applicable.
3. Hyperlink to your specific registration (must be publicly accessible and will be checked): not applicable.

## Guarantor

Nicholas Aderinto.

## Data availability statement

No new datasets were generated for this review.

## Provenance and peer review

Not commissioned, externally peer-reviewed.
